# Control of excitatory hierarchical circuits by parvalbumin-FS basket cells in layer 5 of the frontal cortex: insights for cortical oscillations

**DOI:** 10.1152/jn.00778.2018

**Published:** 2019-04-17

**Authors:** Yasuo Kawaguchi, Takeshi Otsuka, Mieko Morishima, Mika Ushimaru, Yoshiyuki Kubota

**Affiliations:** ^1^Division of Cerebral Circuitry, National Institute for Physiological Sciences, Okazaki, Japan; ^2^Department of Physiological Sciences, SOKENDAI (Graduate University for Advanced Studies), Okazaki, Japan; ^3^Department of Experimental Therapeutics, Institute for Advancement of Clinical and Translational Science, Kyoto University Hospital, Kyoto, Japan

**Keywords:** fast-spiking cell, frontal cortex, gamma oscillation, parvalbumin, pyramidal cell subtype

## Abstract

The cortex contains multiple neuron types with specific connectivity and functions. Recent progress has provided a better understanding of the interactions of these neuron types as well as their output organization particularly for the frontal cortex, with implications for the circuit mechanisms underlying cortical oscillations that have cognitive functions. Layer 5 pyramidal cells (PCs) in the frontal cortex comprise two major subtypes: crossed-corticostriatal (CCS) and corticopontine (CPn) cells. Functionally, CCS and CPn cells exhibit similar phase-dependent firing during gamma waves but participate in two distinct subnetworks that are linked unidirectionally from CCS to CPn cells. GABAergic parvalbumin-expressing fast-spiking (PV-FS) cells, necessary for gamma oscillation, innervate PCs, with stronger and global inhibition to somata and weaker and localized inhibitions to dendritic shafts/spines. While PV-FS cells form reciprocal connections with both CCS and CPn cells, the excitation from CPn to PV-FS cells exhibits short-term synaptic dynamics conducive for oscillation induction. The electrical coupling between PV-FS cells facilitates spike synchronization among PV-FS cells receiving common excitatory inputs from local PCs and inhibits other PV-FS cells via electrically communicated spike afterhyperpolarizations. These connectivity characteristics can promote synchronous firing in the local networks of CPn cells and firing of some CCS cells by anode-break excitation. Thus subsets of L5 CCS and CPn cells within different levels of connection hierarchy exhibit coordinated activity via their common connections with PV-FS cells, and the resulting PC output drives diverse neuronal targets in cortical layer 1 and the striatum with specific temporal precision, expanding the computational power of the cortical network.

## INTRODUCTION

Recent progress in research on the neuronal circuit of frontal cortex has provided a new insight for the interactions of specific cell types within the cortex, with implications for the mechanisms that generate cortical oscillatory activities that play an important role in cognitive functions. We will first review the findings on the local circuits of frontal cortex and then discuss their implications for understanding the mechanisms underlying cortical oscillations.

The neocortex contains morphologically distinct neuronal subpopulations, and the size and density of each of these neuronal subpopulations differ between cortical layers (Jones 1984). Pyramidal cells (PCs), the principal neurons that provide excitatory output from the cortex, are diverse even within each layer in their extracortical targets as well as molecular expression patterns (Tasic et al. 2016), allowing individual layers to have multiple parallel output circuits.

**Fig. 1.**
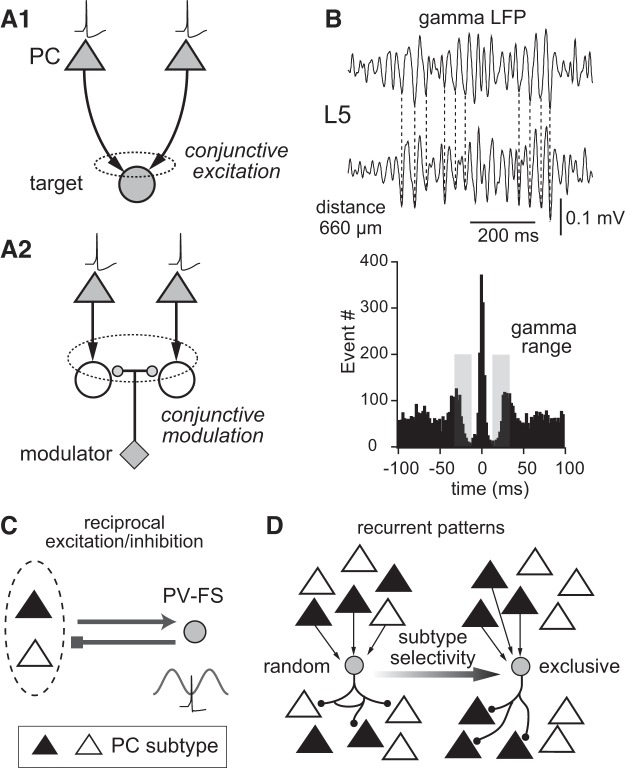
Synchronous firing of diverse subtypes of pyramidal cells (PCs). *A*: actions of synchronously firing PCs to a common target structure in models. *A1*: convergent pathways to a single cell to increase firing probability. *A2*: independent serial pathways from different PCs, with the target cells being modulated by a common input (modulator). *B*: gamma local field potentials (LFPs) recorded from 2 sites within layer 5 (L5) (distance: 660 µm). Wave correlation: 0.75. *Bottom*: cross-correlogram. *C*: Reciprocal connections between PCs and parvalbumin fast-spiking (PV-FS) cells, as a candidate model for gamma oscillation induction. *D*: possible connection patterns of PV-FS cells with PC subtypes. *B*: adapted from Ushimaru and Kawaguchi (2015).

Parallel output circuits play an important role in integrative function of the frontal cortex (Fuster 2015; Miller and Cohen 2001). Some cortico-recipient regions, including neocortical and striatal targets, receive excitation simultaneously from multiple layers, including those from PCs in layer 2/3 (L2/3), layer 5 (L5), and layer 6 (L6) (Markov et al. 2014; Rockland and Pandya 1979; Wilson 1987). Similarly, PCs in both L5 and L6 project to the thalamus (Jones 2001; Usrey and Sherman 2019). Thus each cortico-recipient brain region receives a defined set of afferent drives from multiple neuron subtypes located in one or more cortical layers. These parallel cortical information streams provide the target neuron with synchronous inputs that allow for sophisticated and temporally precise computations (Buzsáki 2006). Indeed, synchronous inputs are more efficacious in exciting recipient neurons than asynchronous drives (Fig. 1*A1*). Even when PC outputs are targeted at different cells, the activity of these target cells can still be conjunctively modulated by other common external inputs, as in the case of dopamine in the striatum (Fig. 1*A2*) (Perrin and Venance 2019). Thus the synchronous output from parallel cortical output circuits expands the computation power of downstream target circuits.

Some PCs could be synchronously fired at a phase of cortical oscillations (Wang 2010). Cortical oscillations can be generated intrinsically by local circuits, as in the case of gamma oscillations (Fig. 1*B*), or extrinsically imposed on the cortex via transmission from subcortical structures such as the thalamus, as is the case for spindle waves (Buzsáki 2006). During gamma oscillations, as a result of reciprocal excitatory and inhibitory connections between PCs and parvalbumin fast-spiking (PV-FS) cells (Fig. 1*C*) (Bartos et al. 2007; Cardin et al. 2009; Pouille and Scanziani 2001; Sohal et al. 2009; Tiesinga and Sejnowski 2009), PCs exhibit synchronous rebound firing following phasic inhibition from PV-FS cells (Fisahn et al. 1998; Whittington et al. 2000). Thus the induction of gamma oscillation requires the coordinated activity among inhibitory GABAergic neurons, especially PV-FS cells (Buzsáki and Draguhn 2004; Hasenstaub et al. 2005; Sohal et al. 2009).

As described above, the striatum receives parallel inputs from PCs residing in multiple cortical layers. In L5 of the frontal cortex, there are two major subtypes of corticostriatal cells with different morphological and projection characteristics (Reiner et al. 2003; Wilson 1987). Since GABAergic inhibition is important for synchronous firing of PCs, identifying the connection patterns of specific GABAergic neurons with the two PC subtypes is necessary for understanding how synchronous firing is initiated in specific sets of PCs. Two opposing hypotheses may be proposed for synaptic connectivity of a GABA cell subtype in cortical circuits, including both excitatory inputs and inhibitory outputs: *1*) connections could be completely nonselective with respect to PC subtypes, or *2*) connectivity may be exclusively subtype dependent (Fig. 1*D*). To understand the circuit mechanisms governing synchronous firing of diverse groups of PCs, we analyzed the activity of L5 PC subtypes and PV-FS cells during cortical oscillations and identified the excitatory and reciprocal inhibitory connections among them in rat secondary motor cortex (M2; rostral part of medial agranular cortex) (Brecht et al. 2004; Ueta et al. 2014). Here, we first outline the rules governing synaptic connectivity of PV-FS cells and PC subtypes and then discuss the circuit mechanisms based on the synaptic connectivity that induce synchronous firing in specific subsets of PCs projecting to layer 1 (L1) and the striatum. These discussions will shed light on the circuit mechanism of simultaneous firing in a specific group of cortical cells.

## PROJECTION-SPECIFIC SUBTYPES OF L5 PCs IN RAT FRONTAL CORTEX

**Fig. 2.**
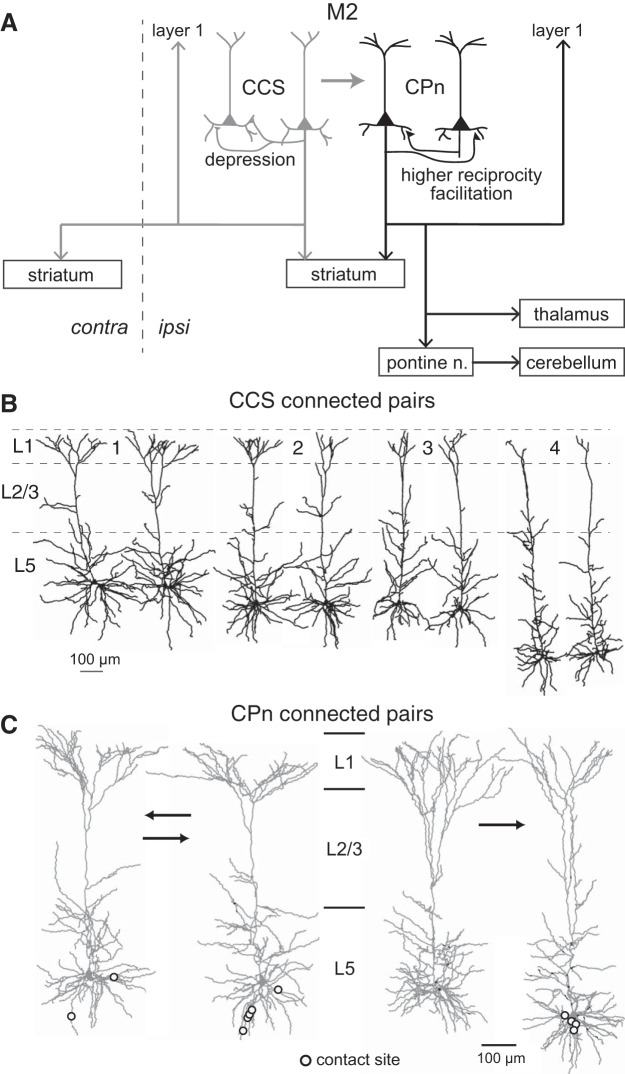
Connectivity and morphological features of 2 subtypes of pyramidal cells (PCs) in L5 of the frontal cortex. *A*: projection patterns and synaptic connections of crossed-corticostriatal (CCS) and corticopontine (CPn) cells; pontine n., pontine nuclei. *B*: Dendritic reconstructions of 4 pairs of synaptically connected CCS cells. Morphologies of 4 pairs of synaptically connected neurons. Note the comparable dendritic patterns between the CCS cells in each pair. *C*: 2 pairs of synaptically connected CPn cells. *Left*: a pair of reciprocally connected cells. *Right*: a pair of cells that are connected unidirectionally. Open circles indicate contact sites. *A*: adapted from Morishima et al. (2011) and Morishima and Kawaguchi (2006). *B*: adapted from Morishima and Kawaguchi (2006). *C*: adapted from Morishima et al. (2011).

In rat frontal cortex, L5 contains two major projection-specific subtypes of PCs: those projecting to the ipsilateral pontine nuclei [corticopontine (CPn) cells], and those projecting to the contralateral frontal cortex [commissural (COM) cells]. The CPn cells correspond to pyramidal tract cells, whereas the COM cells belong to another PC class, intratelencephalic cells (Reiner et al. 2003; Wilson 1987). Both subtypes additionally project to the ipsilateral striatum. These subtypes differ in both electrophysiological properties and dendritic morphologies (Hattox and Nelson 2007; Morishima and Kawaguchi 2006; Otsuka and Kawaguchi 2008). Some COM cells, namely, crossed-corticostriatal (CCS) cells, innervate the striatum bilaterally (Fig. 2*A*) (Morishima and Kawaguchi 2006; Wilson 1987). In addition to their commissural projections, CCS cells innervate ipsilateral frontal and other cortical areas, whereas CPn cells send dense long-distance axon collaterals to the thalamus and ipsilateral frontal cortical areas but project less densely to ipsilateral nonfrontal cortical areas (Hirai et al. 2012; Ueta et al. 2013). Thus the two subtypes of PCs are distinct in their subcortical and corticocortical projection patterns.

One target of PC projections is PCs themselves. In general, PCs of the same subtype are synaptically connected to each other, some in a reciprocal fashion (Morishima and Kawaguchi 2006; Morishima et al. 2011; Otsuka and Kawaguchi 2008, 2011). There are some differences, however, and CPn-to-CPn connections exhibit greater reciprocity in synaptic connections and more short-term synaptic facilitation than found for CCS-to-CCS connections (Morishima et al. 2011). Furthermore, CCS cells innervate CPn cells only unidirectionally (Morishima and Kawaguchi 2006) (Fig. 2*A*). Thus CPn and COM/CCS cells constitute two distinct excitatory subnetworks within L5 that are connected unidirectionally from COM/CCS to CPn networks (Kawaguchi 2017).

Interestingly, synaptically connected pairs of CCS cells share similar morphological features in their apical tufts and basal dendrites (Fig. 2*B*) (Morishima and Kawaguchi 2006). While the connection probabilities between the L5 PCs are low (~0.1), when connections do occur, they typically involve multiple synaptic contacts onto several different dendritic branches of the same postsynaptic PC (Fig. 2*C*) (Morishima and Kawaguchi 2006; Morishima et al. 2011). These findings strongly suggest that synaptic connectivity between the PCs is highly specific, even for PCs with the same projection subtypes, allowing for the establishment of multiple subnetworks. These networks in L5 of the frontal cortex have two hierarchy levels (from COM/CCS to CPn) and exhibit different patterns of synaptic reciprocity and temporal synaptic dynamics in each level.

## COORDINATED OSCILLATORY ACTIVITY OF PV-FS, CCS, AND CPN CELLS

**Fig. 3.**
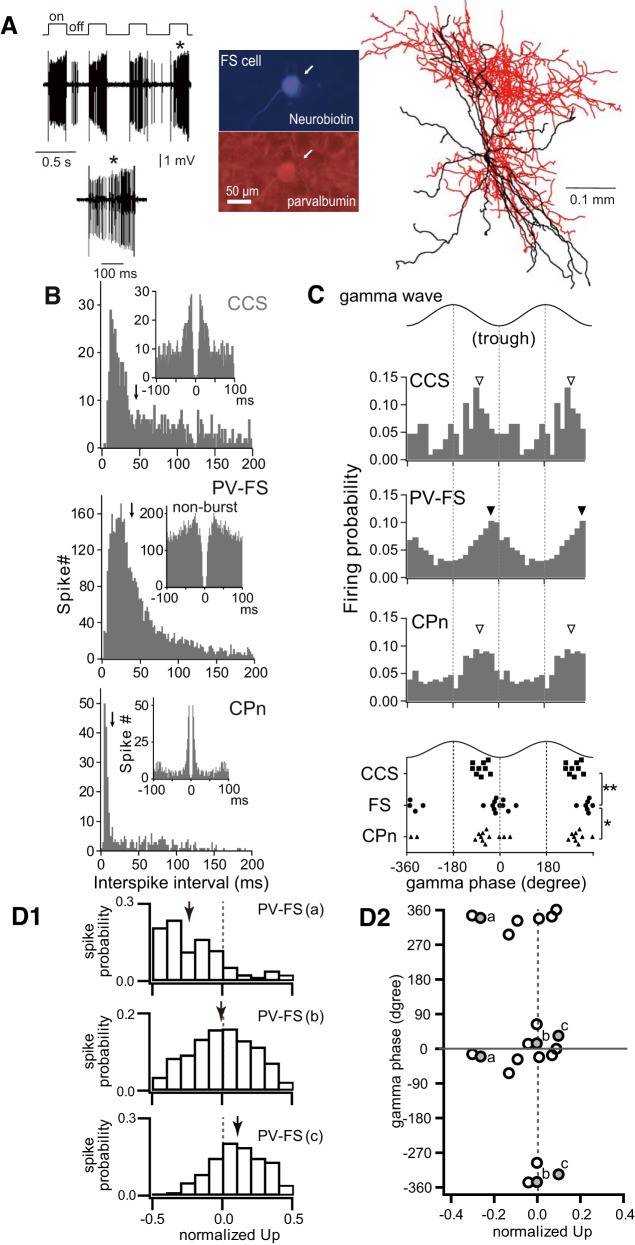
In vivo firing characteristics of parvalbumin fast-spiking (PV-FS), crossed-corticostriatal (CCS), and corticopontine (CPn) cells. *A*: high-frequency firing in a PV-FS cell during juxtacellular depolarizations (on) for neurobiotin labeling in vivo. Center, A neurobiotin-labeled FS cell positive for parvalbumin. Right, reconstruction of the FS cell. The soma and dendrites are shown in black, and the axon in red. *B*: distributions of interspike intervals <200 ms for CCS, FS, and CPn cells. Arrow, median. *Inset*: spike autocorrelogram. *C*: spike distributions of gamma-wave modulated FS, CCS, and CPn cells at the “Up-state” of slow oscillations. Arrowhead, mean firing phase (filled, FS; open, CCS and CPn). Cycles are double-plotted for clarity. Bottom, firing phases of modulated CCS, FS and CPn cells. Phase distributions of FS cells are different from those of CCS cells (**P < 0.01; 2-sample Watson-Williams test) and CPn cells (*P < 0.05). *D1*: spike distributions at Up-states. Firing distributions of 3 FS cells are shown, as follows: *top*: biased to early Up-states; *middle*: not biased to either early or late Up-states; *bottom*: biased to late Up-states. Arrows: median values. *D2*: relationship between Up firing time (abscissa) and gamma phase (ordinate) in L5 PV-FS cells. a, b, and c correspond to the FS cells shown in *D1*. Cycles are double-plotted for clarity. *A*: adapted from Puig et al. (2008). *B*–*D*: adapted from Ushimaru and Kawaguchi (2015).

The firing pattern of GABAergic PV-FS cells has been studied using in vivo juxtacellular recording and neurobiotin labeling followed by post hoc immunohistochemistry for PV (Fig. 3*A*) (Puig et al. 2008). In response to depolarization, PV-FS cells exhibit significantly shorter action potentials and less frequency adaptation during high-frequency burst firing, than typical for PCs. CCS and CPn cells can be identified in vivo via antidromic stimulation from the contralateral striatum and the ipsilateral pontine nuclei, respectively (Ushimaru and Kawaguchi 2015). Furthermore, CPn cells exhibit more rapid doublet firing during initial depolarization than typical of FS or CCS cells (Fig. 3*B*) (Saiki et al. 2018; Ushimaru and Kawaguchi 2015). CCS and CPn cells generate spikes at similar phases of gamma waves during the depolarized “Up-states” of slow cortical oscillations (~1 Hz). By contrast, the firing of FS cells peaks at a later phase of gamma (Fig. 3*C*), consistent with a model in which PV-FS cells are excited by PCs to provide feedback GABAergic inhibition followed by rebound excitation in PCs during the subsequent gamma cycle. Furthermore, different subpopulations of PV-FS cells exhibit activity preferentially at specific time points during slow-wave Up-states (Fig. 3*D*), suggesting that individual FS cells may coordinate synchronous output of subgroups of CCS and CPn cells during specific phases of the slow wave.

## TARGET DIVERSITY AND DOMAIN-DEPENDENT SYNAPTIC STRENGTH OF PV-FS CELL INHIBITIONS

**Fig. 4.**
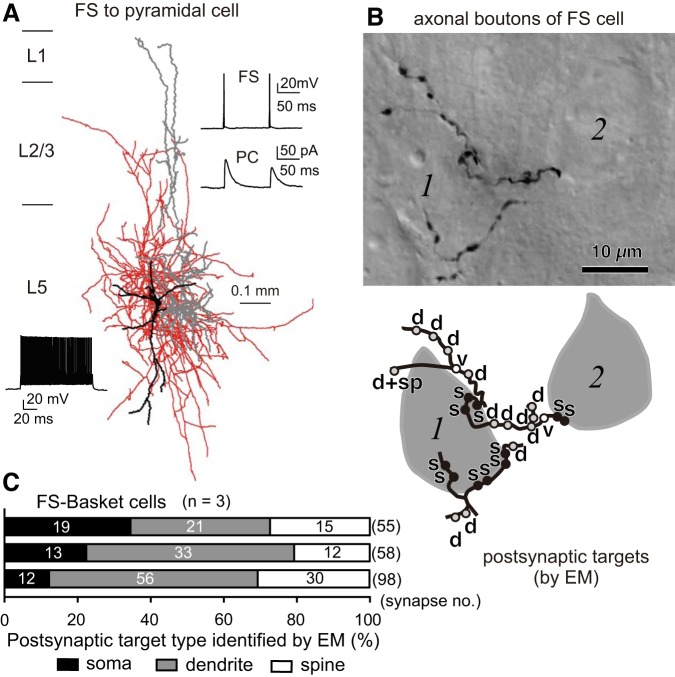
Axonal innervation patterns of parvalbumin fast-spiking (PV-FS) cells. *A*: inhibitory connections from a FS cell to a pyramidal cell (PC). Presynaptic FS cell, dendrites/soma in black and axons in red; postsynaptic PC, dendrites/soma in gray. *Top right inset*: successive inhibitory postsynaptic currents (IPSCs) in the PC in response to FS cell spikes at a 100-ms interval. *Bottom left inset*: nonadaptive firing of high frequency in the FS cell in response to a depolarizing current pulse (200 pA, 1 s). The IPSCs were recorded as outward currents at −40 mV. *B*: boutons of a FS basket cell and their appositions on 2 unstained somata (1, 2) observed with differential interference contrast. *Bottom*: schematic representation of the axon collaterals and boutons shown at the *top*. Postsynaptic targets identified with electron microscopic observation: s, soma; d, dendritic shaft; sp, spine; d + sp, a bouton making 2 synapses on a dendritic shaft and a spine; v, synaptic vesicles were observed, but the junction could not be identified. *C*: innervation patterns of 3 PV-FS basket cells revealed by electron microscopic observations. *A*: adapted from Morishima et al. (2017). *B*: adapted from Karube et al. (2004). *C*: adapted from Kawaguchi and Kubota (1998).

PV-FS cells can be divided into morphological subgroups, including basket cells, in which innervation to PC somata is found at the light microscopic level (Fig. 4, *A* and *B*), and chandelier cells, which selectively target axon initial segments of PCs (Fariñas and DeFelipe 1991; Kawaguchi 1995; Kawaguchi and Kubota 1993; Somogyi 1977). Electron microscopic studies reported that while some axon terminals derived from PV-FS basket cells make synapses onto the somata of pyramidal cells, most innervate dendritic shafts and spines (Karube et al. 2004; Kawaguchi and Kubota 1998; Kubota 2014, 2016). (Fig. 4, *B* and *C*).

**Fig. 5.**
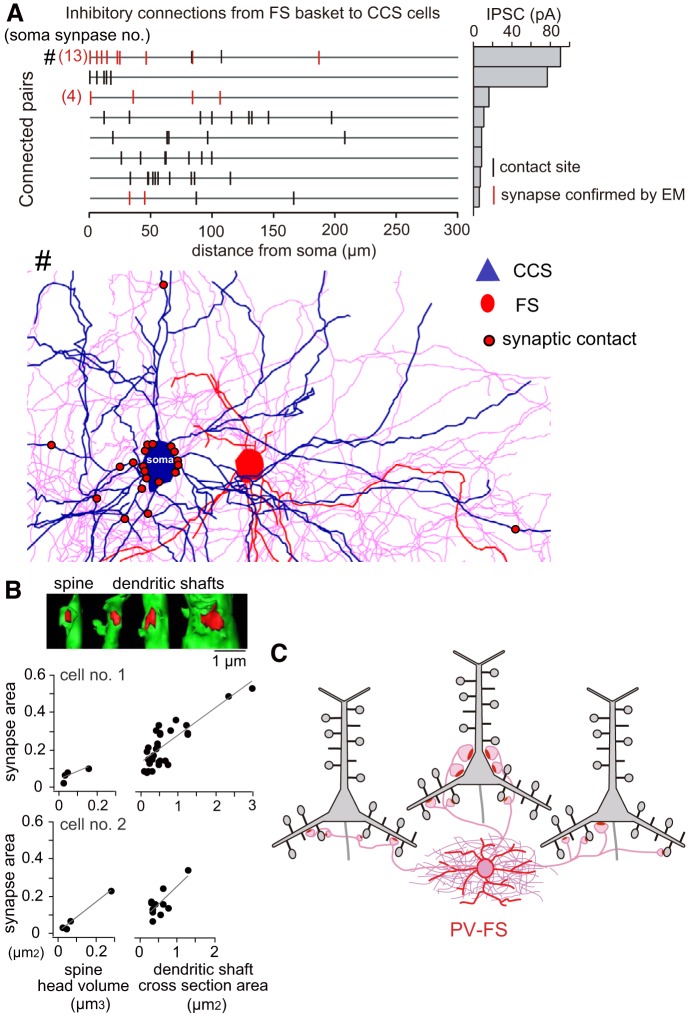
Diverse innervation domains and synaptic junction areas of fast-spiking (FS) cell axons. *A*: distribution of putative synaptic contacts (black bars) made by single FS basket cells with somato-dendritic membrane of 8 connected crossed-corticostriatal (CCS) cells. Contacts confirmed by electron microscopy are shown by red vertical lines, including those with both dendritic shafts and spines. *Right*: the corresponding averaged inhibitory postsynaptic current (IPSC) peak amplitude. The IPSCs were recorded as inward currents at −65 mV with the potassium-based pipette solutions containing 117 mM potassium methylsulfate and 15 mM potassium chloride. *Bottom*: reconstruction of inhibitory synaptic contact sites (red circle) in a pair of FS and CCS cells with 13 somatic innervations (#). *B*: increase in synaptic junction area of FS cell axons with the size of postsynaptic dendritic shafts and spines. *Top*: reconstructions of postsynaptic domains innervated by the FS cell. Postsynaptic target shown in green and synaptic junction area in red. *C*: diversity of postsynaptic structures. Combination patterns of target structures (soma, dendritic shafts, and spines) innervated by individual FS basket cells are diverse among the postsynaptic PCs (CCS cells). *A*–*C*: adapted from Kubota et al. (2015).

The postsynaptic domains of CCS cells targeted by each PV-FS basket cells are diverse, from somata and proximal dendrites of some CCS cells to the dendritic shafts and spines of other CCS cells, as revealed by paired whole cell recordings with post hoc reconstructions of dendrites and axons of recorded cells and electron microscopic observation (Fig. 5) (Kubota et al. 2015). This suggests that PV-FS cells act as classic basket cells for some PCs by targeting somata but as dendrite-targeting cells for other PCs. As expected, the synaptic current in FS to CCS connections is largest when FS cells target somata (Fig. 5). Interestingly, the synaptic junctional areas of PV-FS synapses are positively correlated with the diameter of postsynaptic dendrites (Fig. 5) (Kubota et al. 2015), a correlation also observed for inhibitory connections from PV-FS cells onto spiny projection cells in the striatum (Kubota and Kawaguchi 2000). Since the synaptic current correlates with the junctional area (Nusser et al. 1997), these results suggest selective tuning of PV-FS synaptic strength according to the electrotonic structure of the postsynaptic CCS dendritic compartment.

By applying both the morphological parameters of PCs, including dendritic branching patterns and dendritic shaft/spine sizes, and the physiological parameters of inhibitory synaptic currents to computational models, we showed that inhibition of a spine can cause selective local shunting of excitatory inputs to the spine (Kubota et al. 2015). In accordance with this, GABAergic cells exert compartmentalized control over postsynaptic Ca^2+^ signals within individual dendritic spines (Chiu et al. 2013). These results indicate that PV-FS cells exert diverse domain-selective inhibitions among the postsynaptic PCs.

**Fig. 6.**
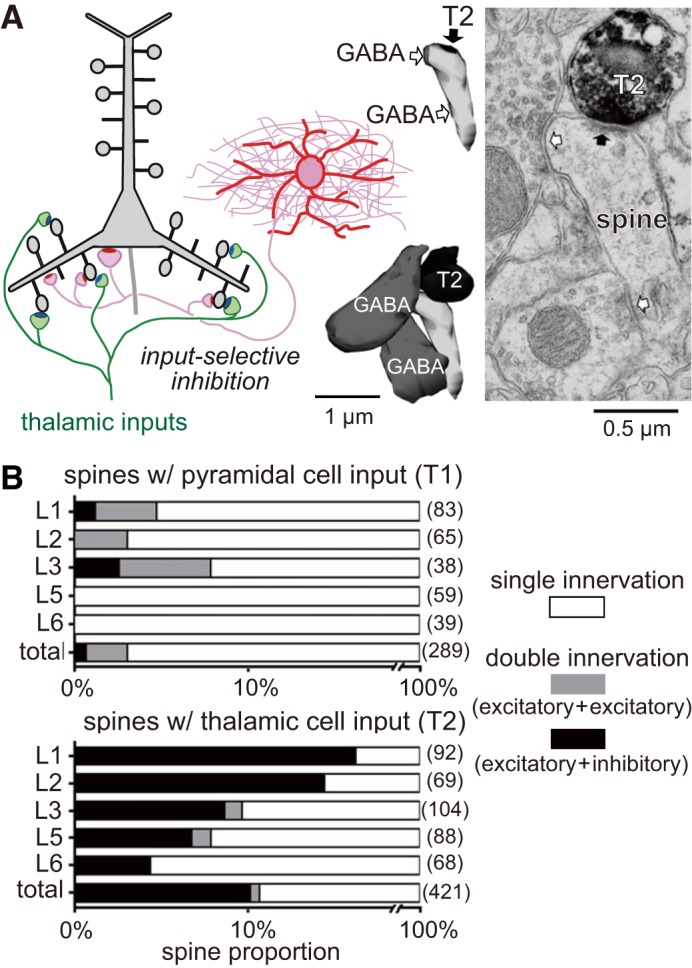
Innervation selectivity of GABAergic cells to pyramidal cell spines. *A*: innervation of a specific type of spines by GABAergic cells. Some spines receiving inputs from the thalamus (T2), but not from the pyramidal cells, are innervated by GABAergic cell axons. *B*: proportion of spines with double innervations. *Top*: spines with inputs from pyramidal cells. *Bottom*: spines with inputs from thalamic cells. *A* and *B*: modified from Kubota et al. (2007).

GABAergic inputs from PV-FS basket cells onto spines of PCs may be critical for regulating recurrent excitation in the local cortical or thalamocortical network. About 2–10% of PC spines are coinnervated by GABAergic and glutamatergic terminals, and this occurs more frequently in the superficial than the deeper layers (Kubota et al. 2007). More remarkably, inhibitory inputs selectively target dendritic spines receiving excitatory input from the thalamus (Fig. 6, *A* and *B*), even though thalamic axonal boutons only make up a minority (~0.2 in layer 3 and ~0.12 in layer 5b in M2) of total glutamatergic boutons (Shigematsu et al. 2016). Since L5 PV-FS cells receive direct thalamocortical inputs (Audette et al. 2018; Hay et al. 2018; Shigematsu et al. 2016), the feedforward GABAergic input to spines from PV-FS cells may be critical for selectively regulating a specific portion of thalamocortical drive to the cortical network.

## CONNECTION SPECIFICITY OF L5 PV-FS CELLS WITH PC SUBTYPES

**Fig. 7.**
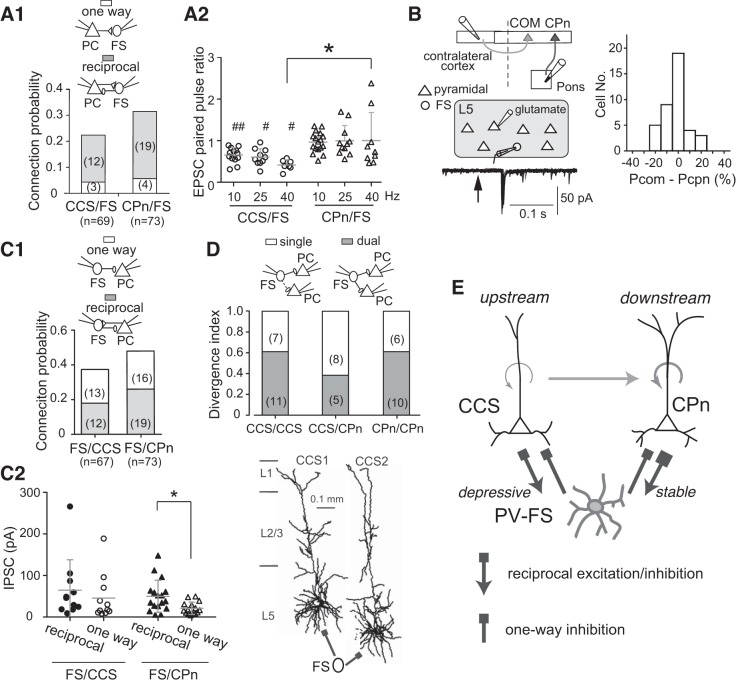
Excitatory and inhibitory connections between layer 5 (L5) pyramidal cells (PCs) and parvalbumin fast-spiking (PV-FS) cells. *A*: excitatory postsynaptic currents (EPSCs) from crossed-corticostriatal (CCS) or corticopontine (CPn) cells to FS cells. *A1*: connection probabilities from CCS and CPn cells. *A2*: paired pulse EPSC ratios between CCS/FS and CPn/FS pairs at 10-, 25-, and 40-Hz stimulation. *B*: excitatory input convergence on FS cells from pyramidal cell subtypes, commissural (COM), and CPn cells. *Left*: EPSC detection in FS cells by glutamate puff stimulations of pyramidal cells (arrow). COM and CPn cells were identified by retrograde tracers. Ten traces are superimposed; right, connection probability differences between COM and CPn cells. *C*: inhibitory outputs from FS cells to L5 PCs. *C1*: inhibitory connection probabilities of FS/CCS and FS/CPn pairs. Gray portion, reciprocally connected. The inhibitory postsynaptic currents (IPSCs) were recorded as outward currents at −40 mV with the potassium-based pipette solutions containing 140 mM potassium gluconate and 7 mM potassium chloride. *C2*: comparison of IPSC amplitudes in FS/CCS and FS/CPn pairs between reciprocal and one-way connections. Circles, CCS cells; triangles, CPn cells. Reciprocally connected FS/CPn pairs have larger IPSCs than pairs with one-way inhibition. *D*: inhibitory output divergence from FS to CCS and CPn cells. Divergence index, proportion of FS cells simultaneously innervating 2 PCs among those FS cells innervating at least 1 of 2 PCs. FS cells innervate PCs, independent of the subtypes. *Bottom*: divergent inhibitory projections from a FS cell to 2 CCS cells. *E*: synaptic connection patterns found between FS cells and PC subtypes. *A*, *C*, *D*, and *E*: modified from Morishima et al. (2017). *B*: modified from Otsuka and Kawaguchi (2013).

CCS and CPn cells innervate PV-FS cells with equal probability, and, in turn, PV-FS cells form reciprocal connectivity with most of those PCs innervating them, as revealed using paired recordings (Fig. 7*A1*) (Morishima et al. 2017). Importantly, most PV-FS cells receive convergent excitation from COM cells (including CCS cells) and CPn cells, as revealed by PV-FS cell recordings paired with alternate glutamate stimulation of COM and CPn cells (Fig. 7*B*) (Otsuka and Kawaguchi 2013). Furthermore, repetitive activation of the two different PC afferents reveals greater short-term depression (i.e., reduced excitatory postsynaptic current amplitudes) by CCS afferents than by CPn afferents (Fig. 7*A2*). Thus, while individual PV-FS cells do not receive preferential excitation from any particular L5 projection subtype, the short-term dynamics of excitatory inputs to PV-FS cells is different depending on the projection subtype of the presynaptic PC. These results suggest that CPn and COM/CCS cells may differentially influence PV-FS cell activity over time.

PV-FS cells make inhibitory connections with CPn and CCS cells with similar probabilities (Fig. 7*C1*) (Morishima et al. 2017). Furthermore, PV-FS cells are reciprocally connected with approximately half of the PCs that they target (Fig. 7*C1*). Importantly, individual PV-FS cells divergently innervate both CPn and CCS cells, as shown with concurrent multiple whole cell recordings (Fig. 7*D*) (Morishima et al. 2017). However, in PV-FS connections with CPn cells, the strength of inhibition is stronger when neurons are reciprocally coupled, relative to unidirectional PV-FS projections (Fig. 7*C2*).

Thus, while L5 PV-FS cells make inhibitory connections nonselectively with both PC projection subtypes, the reciprocal excitatory connections to PV-FS cells show discrete temporal characteristics, with those from CPn cells being more stable for paired pulse stimulation than those from CCS cells; in addition, PV-FS-to-CPn inhibitory synapses are stronger when neurons are reciprocally connected as opposed to when they are only unidirectionally connected (i.e., one-way PV-FS to CPn) (Fig. 7*E*). Furthermore, if one can assume that larger inhibitory postsynaptic currents measured with whole cell recordings reflect somatic synapses while smaller inhibitory postsynaptic currents reflect dendritic inputs (Fig. 5) (Kubota et al. 2015), CPn to FS cell connections might be spatially segregated such that reciprocal connections from CPn neurons target somata, while unidirectional connections favor dendrites (see Fig. 10). The somatic inhibition will promote synchronous rebound firing of CPn cells following GABAergic inhibitory postsynaptic potentials that occur during depolarized states close to spike threshold, potentially establishing reverberatory firing among reciprocally connected populations of PV-FS and CPn cells to establish gamma oscillations (Bartos et al. 2007; Cardin et al. 2009; Pouille and Scanziani 2001; Sohal et al. 2009; Tiesinga and Sejnowski 2009) (see Fig. 10).

The connectivity between PV-FS cells and PCs exhibits additional cortical region-specific variations. For example, in L5 of somatosensory and frontal cortices, PV-FS cells exhibit promiscuous innervation of nearby PCs (Packer and Yuste 2011). By contrast, in L5 of medial prefrontal cortex, PV-FS cells preferentially provide inhibition to thick-tufted subcortically-projecting PCs as opposed to thin-tufted callosally projecting PCs (Lee et al. 2014a). Furthermore, in the hippocampal CA1, PV basket cells provide stronger inhibition to deeper PCs projecting to the amygdala, than to superficial PCs projecting to the medial prefrontal cortex (Lee et al. 2014b; Soltesz and Losonczy 2018). Thus preferential targeting of postsynaptic PCs by PV-FS cells may depend on the cortical area and/or the layer.

Somatostatin (SOM) cells represent another major group of GABAergic cells in the neocortex. L5 low-threshold spike (LTS) cells are a subtype of SOM cells that selectively innervate PC dendrites (Hilscher et al. 2017; Kawaguchi and Kubota 1996; Ma et al. 2006; Morishima et al. 2017). Unlike PV-FS basket cells, individual SOM LTS cells make reciprocal connections selectively with one or the other of the two L5 PC subtypes (Morishima et al. 2017), suggesting that inhibition generated by SOM cells is segregated between two hierarchical levels in L5 (i.e., COM/CCS and CPn). Thus, in contrast to SOM LTS cells, individual PV-FS cells make divergent inhibitory and convergent excitatory connections with both CCS and CPn cells and could regulate PCs at different hierarchy levels simultaneously.

## CONNECTION SPECIFICITY OF ELECTRICALLY COUPLED L5 PV-FS CELLS

**Fig. 8.**
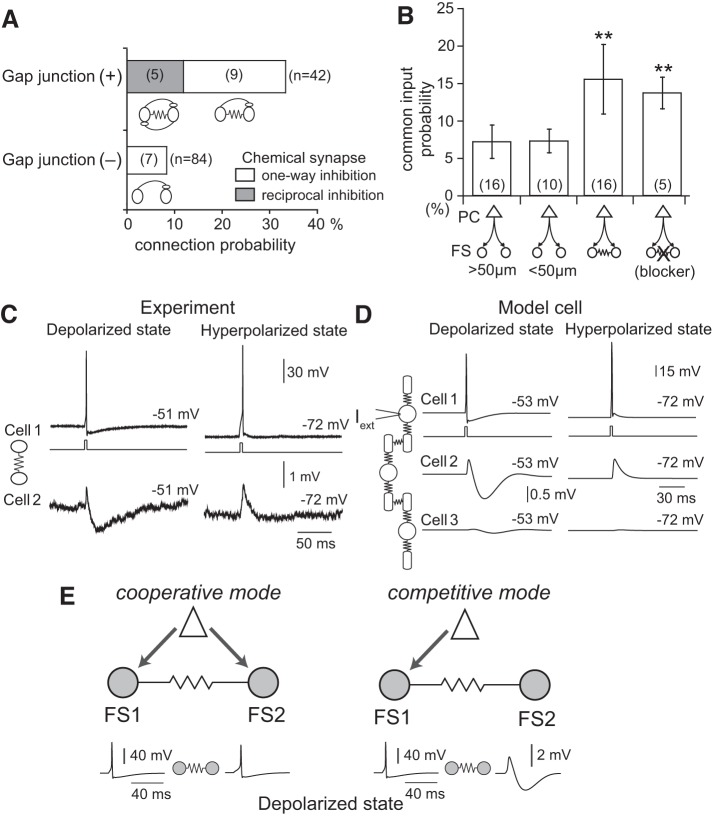
Electrical interactions between fast spiking (FS) cells. *A*: electrical and chemical connection probability between 2 FS cells. Inhibitory connections are more frequently found in electrically coupled pairs. *B*: common input probabilities of 2 FS cells from a pyramidal cells (PCs). *Columns 1 and 2*: electrically unconnected pairs, separated by longer and shorter distance than 50 µm, respectively*. Column 3*: electrically connected pairs. *Column 4*: electrically connected pairs but in the presence of the gap junction blocker. In electrically connected FS/FS pairs as well as those with the gap junction blocker, common input probability is higher than in unconnected pairs. *C*: simultaneous recordings from 2 FS cells connected electrically. *Top*: spike induction by a current pulse in 1 of 2 cells at depolarized (*left*) and hyperpolarized (*right*) states. *Bottom*: postsynaptic voltage responses through gap junction. Membrane potentials were adjusted by DC current injections. *D*: 3 model FS cells connected in series. Current pulses (1-ms duration, Iext) are applied to cell 1 to induce a spike. *E*: 2 types of possible interaction between electrically connected FS cells: cooperative mode and competitive mode. *A*–*E*: modified from Otsuka and Kawaguchi (2013).

PV-FS cells are often electrically coupled through gap junctions (Amitai et al. 2002; Galarreta and Hestrin 1999; Gibson et al. 1999). Electrically connected PV-FS cells are also frequently synaptically connected (Fig. 8*A*) (Galarreta and Hestrin 1999; Hestrin and Galarreta 2005; Hu and Agmon 2015; Otsuka and Kawaguchi 2013). Electrical coupling and inhibitory synaptic connection between cortical GABAergic cells promote their synchronous firing (Connors 2017; Gibson et al. 2005; Merriam et al. 2005). Thus electrical connections among PV-FS cells may promote the induction and maintenance of gamma oscillation (Buhl et al. 2003), although they are not known to be necessary (Neske and Connors 2016).

Electrically connected pairs of PV-FS cells receive excitation from common L5 PCs more frequently compared with noncoupled pairs, as revealed using paired recording of PV-FS cells combined with glutamate stimulation of PCs (Fig. 8*B*) (Otsuka and Kawaguchi 2013). Recordings from a pair of electrically coupled PV-FS cells show that afterhyperpolarizations (AHPs) following action potentials generated in one of the coupled PV-FS cells spread to the other PV-FS cell when the second cell is depolarized but not when it is hyperpolarized (Fig. 8*C*) (Otsuka and Kawaguchi 2013; Vervaeke et al. 2010). A cellular model of electrically connected PV-FS cells based on current-clamp recordings indicates that the AHP will spread primarily to directly coupled cells and will be significantly reduced in cells that are not directly coupled to the primary cell but are coupled via another cell (Fig. 8*D*). Thus the electrical connection between two FS cells supports a dual-mode interaction: cooperation via synchronous firing in response to common excitatory inputs (Fig. 8*E*, *left*) and competition via postspike AHP propagation (Fig. 8*E*, *right*).

## OSCILLATORY ACTIVITY OF PV-FS CELLS GENERATED BY THALAMOCORTICAL INPUTS

**Fig. 9.**
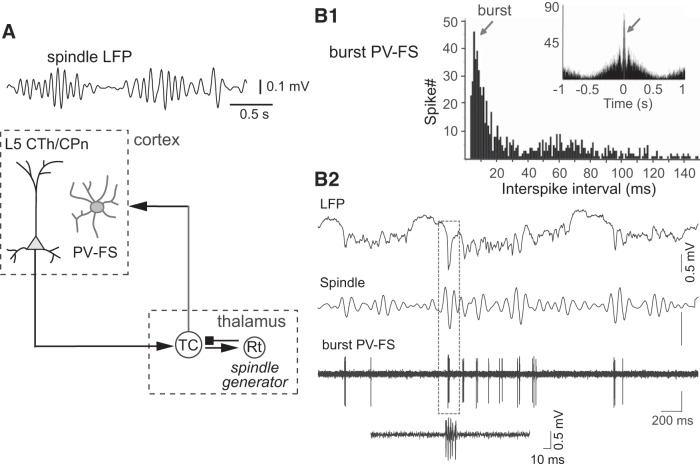
Spindle-wave modulation of parvalbumin fast-spiking (PV-FS) cells. *A*; spindle waves in the frontal cortex. TC, thalamocortical cell; Rt, reticular nucleus cell. *B*: burst PV-FS cell. *B1*: interspike intervals of a burst PV-FS cell. *Inset*: spike autocorrelogram. *B2*: burst firing of a PV-FS cell during spindle (burst PV-FS).Spindle, spindle band digitally filtered from the local field potential (LFP). *A*: modified from Ushimaru et al. (2012). *B*: modified from Puig et al. (2008).

In addition to gamma oscillation generated intracortically during the cortical Up-state, the activity of PV-FS cells is also modulated by oscillatory excitation derived from the thalamus, such as spindle oscillations (7–14 Hz), which also occur during the cortical Up-state (Fig. 9*A*) (Steriade 2006). The spindle rhythm is generated in the thalamic reticular nucleus and transmitted to the cortex via thalamocortical cells (Steriade et al. 1987). The firing of some L5 corticothalamic PCs, as well as thalamocortical cells, is coupled with spindle oscillations (Ushimaru et al. 2012). Similarly, the activity of PV-FS cells is also synchronized with spindle oscillations (Averkin et al. 2016; Puig et al. 2008). Some PV-FS cells fire in high-frequency (~250 Hz) bursts to generate more than two spikes at the troughs of LFP spindles (burst PV-FS cells; Fig. 9*B*). Because PV-FS cells receive excitatory inputs from both local PCs and thalamocortical projections, they participate not only in the generation of gamma oscillations locally but also in the transmission of oscillations generated extrinsically in the thalamus to the cortical local circuit.

## SYNCHRONOUS OUTPUTS FROM L5 HIERARCHICAL EXCITATORY SUBNETWORKS BY PV-FS BASKET CELLS

PV-FS cells are the most numerous GABAergic cells in the frontal cortex and among the best characterized in terms of morphologies, firing patterns, molecular expression, and axonal synaptic structures. While their electrical properties and synaptic transmission have been well described (Hu et al. 2014), how they regulate the activity of diverse networks of PCs is not well understood. To reveal this relationship, it is necessary to determine the rules governing synaptic connectivity between and among PV-FS cells and PC projection subtypes.

As shown in the previous sections, PV-FS cells are well positioned to synchronize the output of the PCs that are located at different hierarchical levels within the L5 excitatory subnetworks (i.e., higher order CCS and lower order CPn cells). This network synchronization most likely requires reciprocal connections between PCs and PV-FS cells in which inhibition from PV-FS cells is targeted to PC somata.

CPn cells exhibit smaller short-term synaptic depression to excitation, as well as reciprocal connectivity with PV-FS cells with preferential innervation by PV-FS cells occurring at the somata of CPn cells. CPn cells frequently discharge doublet spikes during rebound firing (Fig. 3*B*), which, due to the lack of short-term synaptic depression typical of CPn-to-FS synapses, would provide greater excitatory drive back to PV-FS cells than would comparable output from CCS cells. Repeated recurrent excitation between CPn and PV-FS cells would strengthen the connections between these pairs (Lourenço et al. 2014; Vickers et al. 2018). Thus we propose that CPn cells are key contributors to oscillatory firing patterns exhibited by PV-FS cells.

**Fig. 10.**
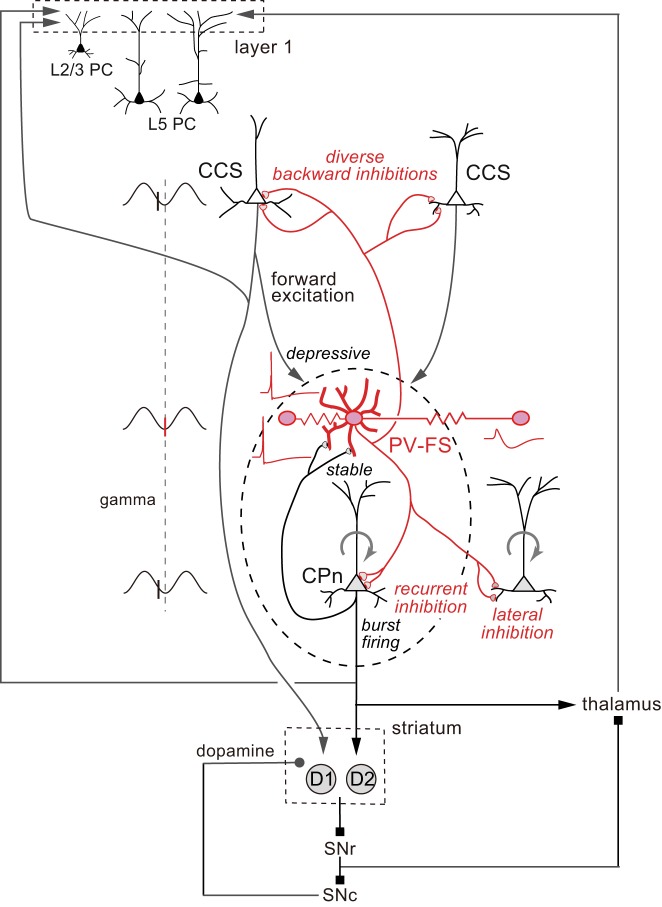
Circuit hypothesis for the induction of synchronous firing in a selected subset of layer 5 (L5) pyramidal cells (PCs) at different hierarchy levels through parvalbumin fast-spiking (PV-FS) basket cells. PV-FS cell receiving excitatory inputs from corticopontine (CPn) cells innervate somata of the same CPn cells, inducing rebound firing at depolarized states by the excitatory/inhibitory reciprocal connections. Repeat of the reciprocal interaction would lead to induction of gamma oscillation. The PV-FS cell inhibit other CPn cells by one-way inhibitory postsynaptic potentials to dendrites (lateral inhibition). Electrically connected PV-FS cells sharing excitation fire simultaneously, but other PV-FS cells without common excitation are suppressed by electrically transmitted postspike AHP (lateral inhibition). Crossed-corticostriatal (CCS) cells do not receive excitation from the CPn cells, but some CCS cells exhibit anode-break excitation following somatic inhibition from the PV-FS cell excited by the CPn cells. Thus PV-FS cells could synchronously activate not only a subset of CPn cells but also CPn and CCS cells at the different hierarchical levels. Common targets of 2 PC subtypes, cortical layer 1 and the striatum, can receive synchronous inputs from a selected subset of CCS and CPn cells. D1, direct cell expressing D1 receptor of dopamine; D2, indirect cell expressing D2 receptor; SNc, substantia nigra pars compacta; SNr, substantia nigra pars reticulata.

For the cortex to generate proper outputs, some PCs are expected to fire synchronously but others to be selectively inhibited. As discussed in the above, our results suggest that CPn cells receive two types of inhibition from FS cells: strong somatic inhibition from reciprocally connected FS cells, and more focused, dendritic inhibition from FS cells with unidirectional connections (Figs. 7 and 10). Thus the activity of other CPn populations would be suppressed as a result of unidirectional dendritic inhibition from PV-FS cells (Fig. 10).

Electrical coupling among PV-FS cells may be also used for synchronous firing of a specific group of PCs. Common excitatory inputs to electrically connected PV-FS cells could induce their synchronous firing, whereas AHP propagation through gap junction suppresses other PV-FS subpopulations (Fig. 10). Thus, via GABAergic synaptic inhibition of unidirectionally targeted CPn cells and through electrical AHP-driven inhibition of other PV-FS cells, PV-FS cells would directly suppress activity in other cortical subnetworks of reciprocally connected CPn and PV-FS cells.

CCS cells that are synaptically connected with each other tend to share similar apical and basal dendritic morphologies (Fig. 2*B*), suggesting that synaptically connected CCS cells will tend to share inputs from afferents targeting the lamina in which the dendrites of these cells show extensive branching. Therefore, CCS cells may comprise serially connected cell groups sharing common inputs. On the other hand, FS cells innervate CCS cells regardless of their dendritic morphologies (Fig. 7*D*). FS cells that fire synchronously with CPn cells innervate the somata of a subpopulation of CCS cells and the dendrites of the other CCS cells (Fig. 10). The former CCS cells could show rebound firing after receiving somatic inhibitory postsynaptic potentials from PV-FS cells, whereas the latter CCS cells would experience less excitatory drive due to dendritic inhibition. Thus the divergent pattern of PV-FS-to-CCS innervation allows for a subnetwork of upstream CCS cells and downstream CPn cells to exhibit synchronous activity paced by feedback inhibition (Isaacson and Scanziani 2011; Tiesinga and Sejnowski 2009).

We speculate that these synchronized CCS and CPn outputs from layer 5 of M2 may have common target cell populations. Both CCS and CPn cells innervate the striatum (Fig. 10). The corticostriatal projections are involved in behavior choice dependent on two temporally different values, expected and outcome values. We have proposed that CCS cells encode a range of selectable actions or states, while CPn cells specify the currently selected action/state (Morita and Kawaguchi 2019; Morita et al. 2012). In other words, one action/state may be represented initially by a group of CCS cells and subsequently by a group of CPn cells receiving input from those CCS cells, with the activity of CPn cells further amplified by faciliatory reciprocal connections among those CPn cells. These two types of cortical inputs can be used for calculation of temporal difference error of values that is represented in firing of dopamine neurons in the substantia nigra (Morita and Kawaguchi 2019; Morita et al. 2012). Corticostriatal synaptic transmission from the two PC subtypes onto direct pathway cells (D1 cells) and indirect pathway cells (D2 cells) in the striatum may be independently modified in a feedback manner by dopamine (Perrin and Venance 2019). Therefore, synchronous firing of appropriate subpopulations of CCS and CPn cells would allow for simultaneous but independent modification of their corticostriatal synapses by dopamine that represents the temporal difference error of values.

L1 does not contain PCs, but PC apical dendrites are innervated by diverse excitatory inputs from the local and other cortical areas as well as the thalamus (Cruikshank et al. 2012; D’Souza and Burkhalter 2017; Guo et al. 2018). Thus it is an important integrative hub for corticocortical and thalamocortical networks (Larkum 2013; Manita et al. 2015). Both CCS and CPn cells send ramified axon collaterals to L1 of M2 as well as other cortices, such as the primary motor and orbitofrontal cortices (Figs. 2*A* and 10) (Hirai et al. 2012; Ueta et al. 2013, 2014). L1 contains the apical dendritic tufts of diverse PCs in L2/3 and L5, as well as several groups of GABAergic cells (Jiang et al. 2015; Kubota et al. 2011; Larkum 2013; Schuman et al. 2019), and is innervated by thalamocortical fibers relaying basal ganglia outputs related to learned motor sequences (Kuramoto et al. 2015; Tanaka et al. 2018). L5 CCS and CPn cells likely innervate multiple targets in L1 and L2 (Hirai et al. 2012). Thus learning of motor sequences may require conjunctive activation of thalamic fibers transmitting basal ganglia output with synchronously active inputs from subsets of CCS and CPn cells innervating L1 (Cho et al. 2011; Suvrathan 2019).

In this perspective, we focused on the local connection patterns of PV-FS cells and two types of output channels from L5 PCs projecting to cortical L1 and striatum that are synchronized by PV-FS cells. The thalamus receives outputs from both the basal ganglia and also from some CPn cells (corticothalamic cells), especially those in upper L5 (L5a), and project back to L1 of the frontal cortex (Figs. 9*A* and 10). Some PV-FS cells are strongly activated by thalamocortical cells in the frontal cortex (Delevich et al. 2015), making PV-FS cells particularly well suited for shaping the activity of local networks of diverse PCs, while also integrating cortico-basal ganglia thalamic feedback to the cortex.

## CONCLUSIONS

Our results demonstrate that L5 PV-FS cells innervate diverse postsynaptic domains, including somata, dendritic shafts, and spines, of the two main L5 PC projection subtypes, namely, CCS and CPn cells. However, individual PV-FS cells exhibit a range of connection specificity with PCs, including preferential targeting of specific surface subdomains, such as dendritic spines that receive thalamocortical input. In addition, PV-FS-to-CPn connections exhibit connectional reciprocity coupled with inhibitory synaptic strength compared with PV-FS-to-CCS connections. In turn, excitation from CPn to PV-FS cells exhibits greater temporal stability than excitatory inputs from CCS cells. Gap junctions between PV-FS cells that share common excitatory inputs form inhibitory subnetworks that reciprocally innervate a diverse network of PC subtypes. The electrical coupling of PV-FS cells synchronizes the output of the inhibitory cells receiving common excitatory input, while providing lateral inhibition via electrically transmitted AHPs.

We propose that the synaptic properties of PV-FS cells, combined with their electrical coupling and selective chemical innervation of diverse postsynaptic domains of PC subtypes, promote synchronous coactivation of subnetworks comprising lower order CPn cells and higher order CCS cells. The resulting coordinated output from the two PC subtypes provides temporally precise drive to the diverse downstream targets in cortical L1 and the striatum, which may play important roles in functional integration of corticocortical and thalamocortical circuits and in outcome value-dependent selection of beneficial actions, respectively.

## GRANTS

The work was supported by Japan Society for the Promotion of Science KAKENHI Grant 17H06311.

## DISCLOSURES

No conflicts of interest, financial or otherwise, are declared by the authors.

## AUTHOR CONTRIBUTIONS

Y. Kawaguchi conceived and designed research; Y. Kawaguchi, T.O., M.M., M.U., and Y. Kubota prepared figures; Y. Kawaguchi drafted manuscript; Y. Kawaguchi, T.O., M.M., M.U., and Y. Kubota edited and revised manuscript; Y. Kawaguchi, T.O., M.M., M.U., and Y. Kubota approved final version of manuscript.
